# Effect of kynurenic acid on enzymatic activity of the DNA base excision repair pathway in specific areas of the sheep brain

**DOI:** 10.1038/s41598-024-66094-x

**Published:** 2024-07-05

**Authors:** Patrycja Młotkowska, Tomasz Misztal, Paweł Kowalczyk, Elżbieta Marciniak

**Affiliations:** Department of Animal Physiology, The Kielanowski Institute of Animal Physiology and Nutrition, Polish Academy of Sciences, Instytucka 3 Str., 05-110 Jabłonna, Poland

**Keywords:** Kynurenic acid, BER pathway, DNA glycosylases, Hypothalamus, Hippocampus, Amygdala, Molecular biology, Neuroscience, Physiology

## Abstract

Relatively low levels of antioxidant enzymes coupled with high oxygen metabolism result in the formation of numerous oxidative DNA damages in the tissues of the central nervous system. Recently, kynurenic acid (KYNA), knowns for its neuroprotective properties, has gained increasing attention in this context. Therefore, our hypothesis assumed that increased KYNA levels in the brain would positively influence mRNA expression of selected enzymes of the base excision repair pathway as well as enhance their efficiency in excising damaged nucleobases in specific areas of the sheep brain. The study was conducted on adult anestrous sheep (n = 18), in which two different doses of KYNA (20 and 100 μg/day) were infused into the third brain ventricle for three days. Molecular and biochemical analysis included the hypothalamus (preoptic and mediol-basal areas), hippocampus (CA3 field) and amygdala (central amygdaloid nucleus), dissected from the brain of sheep euthanized immediately after the last infusion. The results revealed a significant increase *P* < 0.001) in the relative mRNA abundance of *N*-methylpurine DNA glycosylase (MPG) following administration of both dose of KYNA across all examined tissues. The transcription of thymine-DNA glycosylase (TDG) increased significantly (*P* < 0.001) in all tissues in response to the lower KYNA dose compared to the control group. Moreover, 8-oxoguanine (8-oxoG) DNA glycosylase (OGG1) mRNA levels were also higher in both animal groups (*P* < 0.001). In addition, in the hypothalamus, hippocampus and amygdala, AP endonuclease 1 (APE1) mRNA expression increased under both doses of KYNA. Moreover, the both dose of KYNA significantly stimulated the efficiency of 8-oxoG excision in hypothalamus and amygdala (*P* < 0.05–0.001). The lower and higher doses of KYNA significantly influenced the effectiveness of εA and εC in all structures (*P* < 0.01–0.001). In conclusion, the favorable effect of KYNA in the brain may include the protection of genetic material in nerve and glial cells by stimulating the expression and efficiency of BER pathway enzymes.

## Introduction

Base excision repair (BER) is a key DNA repair pathway involved in maintaining genome stability, which has been shown to prevent premature aging, neurodegenerative diseases, and cancer^1^. Enzymes within the BER pathway exhibit substrate specificity for certain types of damaged nucleobases, effectively removing these lesions from the DNA chain. The pathway includes two types of DNA glycosylases: mono- and bifunctional enzymes. The former break the N-glycosidic bond between the damaged base and the DNA phosphodiester backbone, creating an AP site^2^. The resulting AP site is recognized by AP endonuclease 1 (APE1), which cleaves the DNA backbone and forms a single-nucleotide gap flanked by 3′-hydroxyl and 5′-deoxyribosephosphate termini. In contrast, bifunctional DNA glycosylases nick the DNA backbone and create a single-nucleotide gap flanked by either 5′-phosphate and 3′-α,β-unsaturated aldehyde (referred to as β-elimination) or 5′-phosphate and 3′-phosphate residues (known as β,δ-elimination)^3^. The glycosylases responsible for catalyzing β-elimination include 8-oxoguanine (8-oxoG) DNA glycosylase (OGG1) and endonuclease homolog III (NTH1). Subsequently, APE1 cleaves the 3′-α,β-unsaturated aldehyde to generate the 3′-hydroxyl end, which is generated by monofunctional DNA glycosylase. However, APE1 can bypass this step and directly cleave the AP site itself. Enzymes catalyzing β,δ-elimination comprise endonuclease VII-like proteins 1 to 3 (NEIL1 to -3), and the 3′-phosphate formed as a result of their action is cleaved by polynucleotide kinase phosphatase (PNKP)^4,5^. The findings described above have led to the identification of both APE1-dependent and APE1-independent (PNKP-dependent) BER pathways. The end product of BER transformations is a single-nucleotide gap containing a 3′-hydroxyl end, serving as a substrate for DNA polymerase. DNA polymerase β (Pol β) is the primary enzyme involved in the BER pathway, which inserts an intact base to complete the repair process^1,6^. Therefore, endogenous stimulation of BER enzymes activities in the central nervous system (CNS) may be an important mechanism for maintaining genomic integrity in cells and diminishing the risk of various brain pathologies caused by oxidative stress.

In addition to the aforementioned enzymes, N-methylpurine DNA glycosylase (MPG) and thymine-DNA glycosylase (TDG) also play crucial roles in the BER pathway. MPG is responsible for excising damaged bases such as N3-methyladenine (N3-meA), N7-methylguanine (7-meG), N3-methylguanine (N3meG), hypoxanthine, 1,N^6^-ethenoadenine and 8-oxoguanine (8oxoG). Increasing the ratio of MPG to polymerase in both the nucleus and mitochondria has been found to influence the effectiveness of BER^7^. Initially presumed to primarily remove U:G and T:G mismatches, TDG has since been found to glycosylates various mismatched pyrimidine bases, as well as oxidized/halogenated bases. Additionally, TDG plays a role in transcriptional regulation by aiding in the assembly of the coactivator complex and facilitating active DNA demethylation^8^.

Kynurenic acid (KYNA) is neuroactive metabolite of tryptophan, synthesized in the kynurenine pathway, primarily through the transamination of L-kynurenine by kynurenine aminotransferase in the CNS and various peripheral tissues in mammals^9,10^. KYNA acts as neuromodulator, exerting its effects on several types of receptors. It is a high-affinity antagonist at the strychnine-insensitive NR1 site of *N*-methyl-d-aspartic acid receptors (NMDAR), contributing to antidepressant and psychotomimetic effects^11^. It also serves as a modulator in the nicotinic cholinergic system, increasing α4β2 α7-nicotinic acetylcholine receptors (nAChR) expression and, more specifically, by inhibiting nAChRs noncompetitively and voltage independently. Additionally, KYNA binds to α-amino-3-hydroxy-5-methyl-4-isoxazole-propionic acid (AMPA) receptors acting as an agonist of the glutamate receptor and G-protein-coupled receptor (GPR35) in immune cells. Apart from its immune functions, the KYNA-GPR35 compound may exert an inhibitory effect on Ca^2+^ channels in sympathetic neurons and reduce synaptic activity in hippocampal neurons^12,13^. Moreover, in immune and cancer cells, KYNA is an endogenous agonist of the aryl hydrocarbon receptor (AHR). All these receptors have been shown to mediate several important processes in the CNS, including neurodevelopment, plasticity, cognition, behavior, and memory. In addition, evidence suggests that KYNA has antioxidant properties and may act as a scavenger of reactive oxygen species (ROS). It should be noted that brain tissue maintains a particularly high basal metabolic rate to meet its high energy demands, which means that brain cells produce high levels of ROS^14,15^. Interestingly, several factors make the brain additionally susceptible to oxidative damage. Therefore, an imbalance in antioxidant and oxidant processes can pose a major threat to brain tissue. Studies have demonstrated KYNA ability to remove different ROS and reduce the levels of important oxidative damage markers produced by various pro-oxidants in tissue preparations^14^. Taking into account the relationship between neurodegenerative diseases or the aging brain process and impaired functioning of the antioxidant system^15^, as well as the favorable effects of KYNA in various CNS pathologies^16^, expanding knowledge about neuroprotective effects of this tryptophan metabolite is of great importance. According to the available literature, the relationship of KYNA with the expression and activity of the main DNA defense enzymes (BER pathway) in the CNS is not well known. Therefore, our present study aimed to investigate whether elevated KYNA levels in the brain could potentially enhance mRNA expression of specific BER pathway enzymes and improve their efficiency in excising lesioned nucleobases in specific areas of the sheep brain.

## Materials and methods

### Animal management

All procedures were conducted in accordance with the Code of Ethics of the World Medical Association (Declaration of Helsinki), as well as the EU Directive 2010/63/EU on animal experimentation. Approval for the study (no. WAW2/128/2020) was obtained from the Local Ethics Committee affiliated with Warsaw University of Life Sciences, in compliance with the Polish Law for the Animal Care and Use of January 21, 2005 and the Polish Law for Animal Protection (of September 16, 2011). Also presented experiment (design and all procedures) fulfil criteria of ARRIVE guidelines.

Eighteen Polish Longwool sheep (a breed showing reproductive seasonality), aged 1 year and weighing 55 ± 2 kg were used in the experiment. They were bred indoors at the Sheep Breeding Center of the Kielanowski Institute of Animal Physiology and Nutrition, Polish Academy of Sciences (Jablonna near Warsaw, Poland) under natural lighting conditions (52° N, 21° E). The animals were fed twice a daily a diet based on pellet concentrate that followed the recommendations of the National Research Institute of Animal Production in Krakow–Balice, Poland, and the National Institute for Agricultural Research in France^17^. During the experimental period, sheep were kept in individual pens, providing them with visual, olfactory and tactile contact and free access to water and mineral licks.

### Brain surgery—third ventricle (IIIv) cannulation

One month before the experiment, sheep underwent surgical implantation of a cannula into the IIIv of the brain (outer diameter—1.2 mm, position: frontal—31.0 mm), in accordance with the stereotaxic coordinate system for sheep hypothalamus^18^. Implantation was performed under general anesthesia (xylazine: 40 mg/kg body mass, intravenously; xylapan and ketamine: 10–20 mg/kg body mass, intravenously; Bioketan, Vetoquinol Biowet, Pulawy, Poland) through a hole drilled in the skull, in accordance with the procedure described by Traczyk and Przekop^19^. A guide cannula was fixed to the skull with stainless steel screws and dental cement. The external opening of the canal was closed with a stainless-steel cap. After the surgery, ewes were injected for four days with antibiotics (1 g streptomycin and 1,200,000 IU benzylpenicillin; Polfa, Poland) and analgesics (metamizole sodium: 50 mg/animal; Biovetalgin, Biowet Drwalew, Poland, or meloxicam: 1.5 mg/animal; Metacam, Boehringer Ingelheim, Germany). Placement of the cannula in the IIIv was confirmed by cerebrospinal fluid (CSF) outflow during surgery and after slaughter. The sheep used in the present study had a correctly located cannula.

### Experimental design and tissue collection

Experiment was performed in March during the natural anestrous season for this breed of sheep. The animals were randomly divided into three groups (n = 6 each) and infused into the IIIv with Ringer-Locke solution (RLs, control) or with one of two doses of KYNA (Sigma Chemical Co., St Louis, MO, USA) dissolved in RLs. The solubility of KYNA in RL solution and pH was tested earlier, before starting the experiment. The treatment was performed in a series of four 30 min infusions, at 30 min intervals, from 10:00 to 14:00. The doses of KYNA (lower: 4 × 5 μg/60 μL/30 min and larger: 4 × 25 μg/60 μL/30 min) were selected on the basis of scientific literature^20^. All infusions were performed using a BAS Bee microinjection pump (Bioanalytical Systems Inc., West Lafayette, IN, USA) and calibrated 1.0 mL gas-tight syringes. During the treatments, sheep were kept in pairs in the experimental room in comfortable cages, where they will able to lie down and to which they were previously adapted for three days. Immediately after the experiment, sheep were slaughtered after prior pharmacological stunning (xylazine 0.2 mg/kg of body mass and ketamine: 3 mg/kg of body mass, intravenously) and the brains were rapidly removed from the skull. After separation of the ME, each brain was sectioned sagittally into the cerebral hemispheres. The isolated blocks of the hypothalamus (cutting to a depth of 2 mm) were dissected into two parts: preoptic area (POA) and mediol-basal hypothalamus (MBH)^21^, according to the stereotaxic atlas of the ovine brain^18^. The hippocampus was dissected from the medial part of the temporal lobe of the right hemisphere, starting from the floor of the lateral ventricle through ventral and dorsal parts, according to the sheep brain atlas [http://brains.anatomy.msu.edu/brains/sheep/index.html]. Sections measuring about 2–3 mm in length were cut out from the CA3 field of the hippocampus. Subsequently, a block of the right amygdala (AMG) (thought to be linked to negative emotions^22^) covering the central amygdaloid nucleus (thought to be involved in behavioral, autonomic, and endocrine responses^23^) was isolated by a point cut-out to a depth of approximately 2 mm^24^. All tissue cuts were performed on sterile glass plates placed on ice, then the collected structures were immediately frozen in liquid nitrogen and stored at − 80 °C.

### Relative Abundance of mRNA Analysis

Total RNA from POA, MBH, CA3 and AMG tissues was isolated using the NucleoSpin RNA II kit (MachereyNagel, Düren, Germany) 4 × 4500, according to the manufacturer’s protocol. The concentration and purity of isolated RNA were quantified using a NanoDrop ND-1000 spectrophotometer (Thermo Fisher Scientific). RNA integrity was electrophoretically verified on a 1.5% agarose gel stained with ethidium bromide. The TranScriba Kit (A&A Biotechnology, Gdynia, Poland) 10 × 1000 was used to synthesize cDNA according to the manufacturer’s instructions (1 µg of total RNA in a reaction volume of 20 µL). Quantitative polymerase chain reaction (qPCR) was performed using 5 × HOT FIREPol® EvaGreen qPCR Mix Plus (Solis BioDyne, Tartu, Estonia). The PCR amplification mix contained 2 µL of cDNA template, 1 µL of primers (0.5 µL each at 10 pmol/mL), 3 µL of buffer PCR Master Mix and 9 µL of dd H_2_O. The reaction conditions were as follows: initial denaturation at 95 °C for 15 min, denaturation at 95 °C for 15 s, annealing at 60 °C for 20 s, and elongation at 72 °C for 20 s (40 cycles). Specific primers for determining the expression of 8-oxoguanine glycosylase (*OGG1*), N-methylpurine DNA glycosylase (*MPG*), thymine DNA glycosylase (*TDG*) and AP-endonuclease 1 (*APE1*) genes, as well as endogenous control genes: glyceraldehyde-3-phosphate dehydrogenase *(GAPDH)* and peptidylprolyl isomerase C *(PPIC)* were designed using Primer3 software (The Whitehead Institute, Boston, MA, USA) and are listed in Table 1. Amplification specificity was further validated by electrophoresis of the obtained amplicons in a 2% agarose gel and visualized under a UV light camera^25,26^. Data were analyzed with Rotor Gene 6000 v. 1.7 software (Qiagen GmbH, Hilden, Germany) using a comparative quantification option and Relative Expression Software Tool, based on the PCR efficiency correction algorithm developed by Pfaffl et al.^27,28^. The expression levels of the tested genes were normalized using geometrical means of the reference genes expression. The endogenous control genes were assayed in each sample to compensate for variation in cDNA concentration and PCR efficiency between individual tubes.

**Table 1:** Specific primers sequences.

Gene	Primers (5′–3′)	Genbank Acc. No	Amplicon size
*MPG*	F: GCTGAGGGCCAGCCAACACCTGC R: CGCCCCTTTACCCACGGAGCCCA	XM_027962019.2	121
*TDG*	F: TAATGGGCAGTGGATGACCC R: TAATGGGCAGTGGATGACCC	XM_027967675.3	128
*OGG1*	F: CTCAGAAATTCCAAGGTGTTC R:CCGCTCCACCAT-GCCAGTG	XM_012099510.5	113
*APE1*	F: GAATGCTGGCTTCACTCCACA R: AAAGGTGTAGGCATACGCCGT	XM_004010390.5	115
*GAPDH*	F: GGGTCATCATCTCTGCACCT R: GGTCATAAGTCCCTCCACGA	NM_001190390.1	131
*PPIC*	F: ACGGCCAAGGTCTTCTTTG R: TATCCTTTCTCTCCCGTTGC	NM_001076910	131

### Enzyme repair activity

The oligonucleotides (40-mer) containing a single 8-oxo-guanine (8-oxoG), 1,*N*^6^-ethenodeoxyadenine (εA) and 3,*N*^4^-ethenodeoxycytosine (εC) at position 20 in the sequence 50-d(GCT ACC TAC CTA GCG ACC TXC GAC TGT CCC ACT GCT CGA)-30, where X indicates lesioned nucleobases, were obtained from Eurogentec Herstal (Herstal, Belgium) or Genset Oligos (Paris, France). The oligonucleotides were 32P-labeled at the 50-end by polynucleotide kinase at an excess of [32P]ATP (3000 Ci/mmol) (Amersham, Little Chalfont, UK). Radiolabeled oligomers were purified from unincorporated radioactivity using Micro Bio-Spin P-30 columns, as described by the manufacturer (Bio-Rad, Hercules, CA, USA). These oligomers were annealed at double-molar excess to complementary oligonucleotides containing T opposite εA, G opposite εC, or C opposite 8-oxoG. Complementary oligodeoxynucleotides were synthesized according to standard procedures using an Applied Biosystems synthesizer (Oligonucleotide Synthesis Laboratory, Institute of Biochemistry and Biophysics, Polish Academy of Sciences). Repair activity of BER pathway enzymes was determined based on the excision efficiency of damaged nucleobases (8-oxoG, εA and εC) using the nicking method, as described in detail by Misztal et al.^24^ and Kowalczyk et al.^29^.

### Statistical analysis

Initially, all data were tested for normality with the Shapiro–Wilk normality test and then grouped into parametric and non-parametric groups. Excision efficiency of damaged nucleobases were analyzed using one-way analysis of variance (STATISTICA, Stat Soft, Tulsa, OK, USA). The post-hoc Tukey test was performed after each analysis. Statistical evaluations of differences in OGG1, MPG, TDG and APE1 mRNA expression in tissues studied between treatment groups were carried out using non-parametric statistics, involving the Kruskal–Wallis test, followed by multiple comparisons of average ranks, then the Mann–Whitney *U* test for particular groups. Differences were considered significant at *P* < 0.05, and all data are presented as a mean ± standard error of the mean (SEM).

### Ethics declarations

All procedures were conducted in accordance with the Code of Ethics of the World Medical Association (Declaration of Helsinki), as well as the EU Directive 2010/63/EU on animal experimentation. Approval for the study (no. WAW2/128/2020) was obtained from the Local Ethics Committee affiliated with Warsaw University of Life Sciences, in compliance with the Polish Law for the Animal Care and Use of January 21, 2005 and the Polish Law for Animal Protection (of September 16, 2011). Also presented experiment (design and all procedures) fulfil criteria of ARRIVE guidelines.

## Results

### Expression and mRNA activity of DNA glycosylases

**Figure 1:**
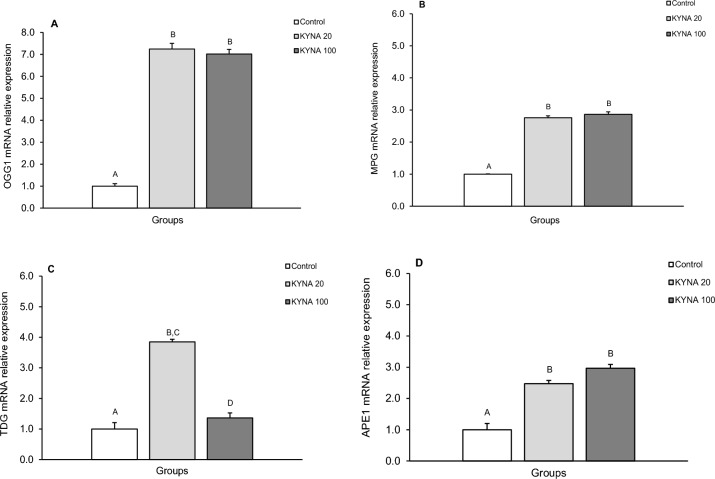
Relative mRNA abundance (mean ± SEM) of 8-Oxoguanine glycosylase (OGG1) (**A**), N-methylpurine-DNA glycosylase (MPG) (**B**), thymine-DNA glycosylase (TDG) (**C**) and AP-endonuclease 1 (APE1) (**D**) in the medio-basal hypothalamus (MBH) of the control group in sheep infused with the lower (KYNA 20–20 μg in total/day) and higher (KYNA 100–100 μg in total/day) dose of kynurenic acid into the third ventricle of the brain. Significance of differences: AB,CD; *P* < 0.001.

**Figure 2:**
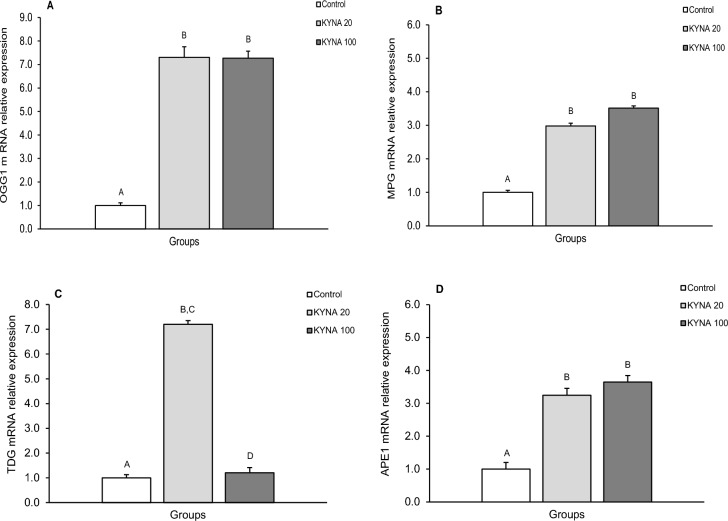
Relative mRNA abundance (mean ± SEM) of 8-Oxoguanine glycosylase (OGG1) (**A**), N-methylpurine-DNA glycosylase (MPG) (**B**), thymine-DNA glycosylase (TDG) (**C**) and AP-endonuclease 1 (APE1) (**D**) in the preoptic area (POA) of the control group in sheep infused with the lower (KYNA 20–20 μg in total/day) and higher (KYNA 100–100 μg in total/day) dose of kynurenic acid into the third ventricle of the brain. Significance of differences: AB,CD; *P* < 0.001.

**Figure 3:**
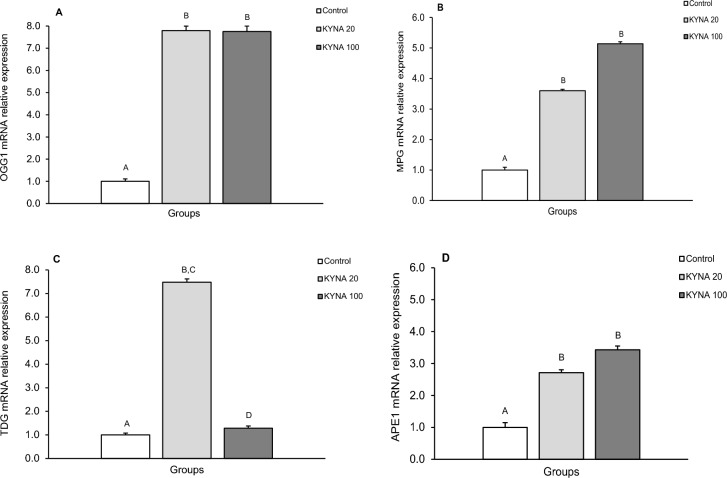
Relative mRNA abundance (mean ± SEM) of 8-Oxoguanine glycosylase (OGG1) (**A**), N-methylpurine-DNA glycosylase (MPG) (**B**), thymine-DNA glycosylase (TDG) (**C**) and AP-endonuclease 1 (APE1) (**D**) in the hippocampal field (CA3) of the control group in sheep infused with the lower (KYNA 20–20 μg in total/day) and higher (KYNA 100–100 μg in total/day) dose of kynurenic acid into the third ventricle of the brain. Significance of differences: AB,CD; *P* < 0.001.

**Figure 4:**
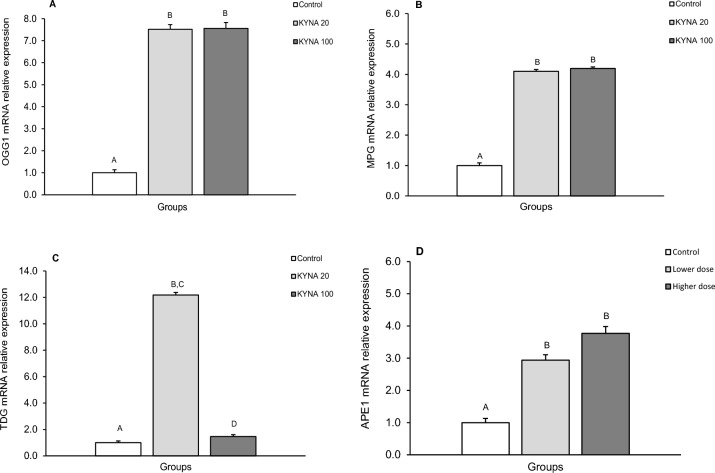
Relative mRNA abundance (mean ± SEM) of 8-Oxoguanine glycosylase (OGG1) (**A**), N-methylpurine-DNA glycosylase (MPG) (**B**), thymine-DNA glycosylase (TDG) (**C**) and AP-endonuclease 1 (APE1) (**D**) in the amygdala (AMG) of the control group in sheep infused with the lower (KYNA 20–20 μg in total/day) and higher (KYNA 100–100 μg in total/day) dose of kynurenic acid into the third ventricle of the brain. Significance of differences: AB,CD; *P* < 0.001.

The expression of gene transcripts for all DNA glycosylases examined (OGG1, MPG, and TDG) and APE1 was detected in all tissues of interest. The relative abundances of these transcripts in the mediol-basal hypothalamus (MBH), preoptic area (POA), hippocampal CA3 region, and amygdala (AMG) in all treatment groups are depicted in Figs. 1, 2, 3, 4.

The level of OGG1 mRNA expression (Figs. 1A, 2A, 3A, 4A) significantly increased (*P* < 0.001) in groups infused with both the lower and higher doses of KYNA compared to the control group. However, there was no significant differences in the level of OGG1 transcripts between the two KYNA-treated groups.

The relative expression of MPG mRNA (mean ± SEM) also significantly increased (*P* < 0.001) in response to both doses of KYNA in all tissues examined compared to the control group (Figs. 1B, 2B, 3B, 4B). While MPG mRNA expression levels were similar in response to KYNA in the MBH, POA and AMG (Figs. 1B, 2B, 4B), the higher dose elevated the expression in the CA3 (*P* < 0.001) more pronouncedly compared to the lower dose of KYNA (Fig. 3B).

TDG mRNA expression increased (*P* < 0.001) in response to the lower KYNA dose in all tissues examined (Figs. 1C, 2C, 3C, 4C) compared to the control group and to the group receiving the higher KYNA dose (*P* < 0.001). In the MBH, POA and CA3, there was a 4 to sevenfold increase in the MPG transcript abundance (Figs. 1C, 2C, 3C), while in the AMG, the increase was considerably higher (12-fold), marking the highest response among the structures examined (Fig. 4C).

A consistent pattern of APE1 mRNA expression was recorded in response to KYNA infusion, showing significant increases (*P* < 0.001) in the MBH, POA, CA3 and AMG with both KYNA doses compared to the control group (Figs. 1D, 2D, 3D,4D). Moreover, across all analyzed structures, the abundance of APE1 transcript increased more markedly in response to the higher dose of KYNA relative to the lower dose.

**Figure 5:**
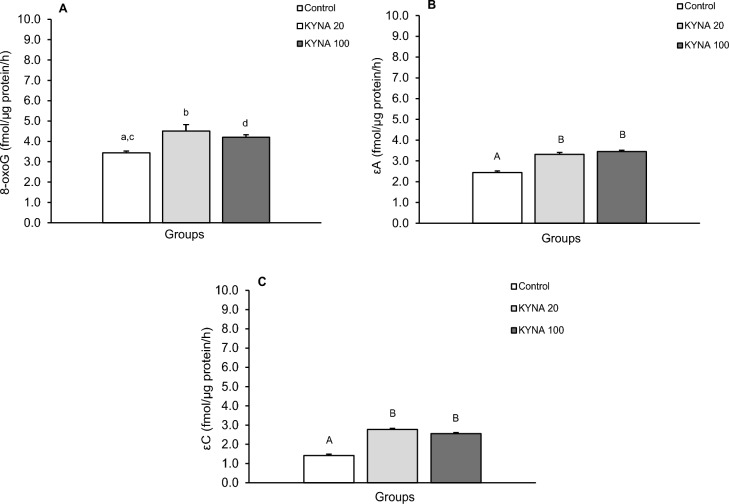
Repair activities for 8-oxoguanine (8-oxoG) (**A**), 1,N6-ethenoadenine (εA) (**B**) and 3,N4-ethenocytosine (εC) (**C**) in the medio-basal hypothalamus (MBH) of sheep infused with the lower (KYNA 20–20 μg in total/day) and higher (KYNA 100–100 μg in total/day) dose of kynurenic acid into the third ventricle of the brain. Significance of differences: cd, *P* < 0.05; ab, *P* < 0.01; AB, *P* < 0.001.

**Figure 6:**
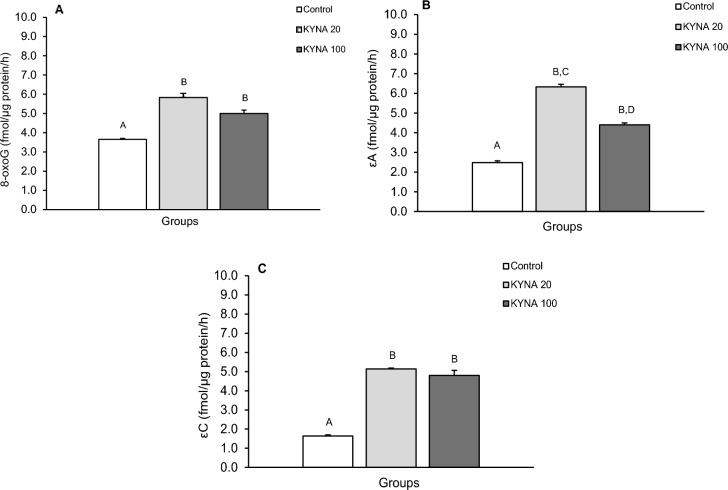
Repair activities for 8-oxoguanine (8-oxoG) (**A**), 1,N6-ethenoadenine (εA) (**B**) and 3,N4-ethenocytosine (εC) (**C**) in the preoptic area (POA) of sheep infused with the lower (KYNA 20–20 μg in total/day) and higher (KYNA 100–100 μg in total/day) dose of kynurenic acid into the third ventricle of the brain. Significance of differences: AB,CD, *P* < 0.001.

**Figure 7:**
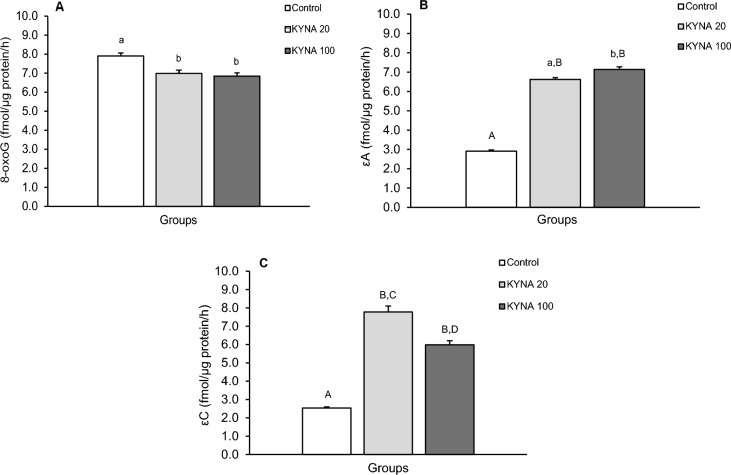
Repair activities for 8-oxoguanine (8-oxoG) (**A**), 1,N6-ethenoadenine (εA) (**B**) and 3,N4-ethenocytosine (εC) (**C**) in the hippocampal field (CA3) of sheep infused with the lower (KYNA 20–20 μg in total/day) and higher (KYNA 100–100 μg in total/day) dose of kynurenic acid into the third ventricle of the brain. Significance of differences: ab, *P* < 0.01; AB, CD, *P* < 0.001.

**Figure 8:**
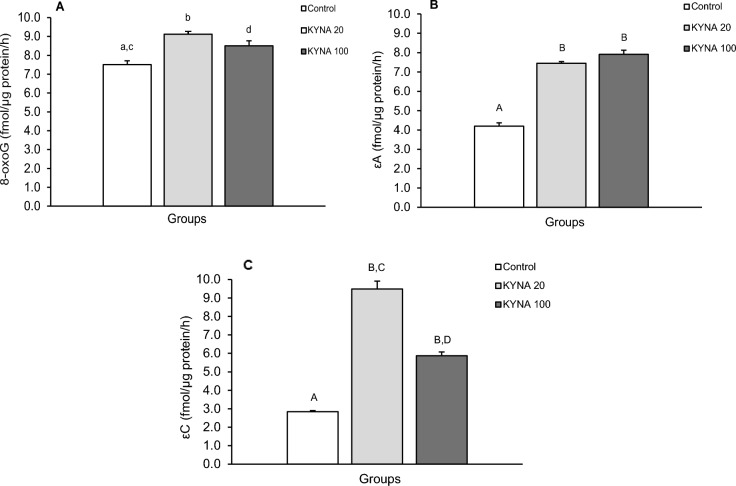
Repair activities for 8-oxoguanine (8-oxoG) (**A**), 1,N6-ethenoadenine (εA) (**B**) and 3,N4-ethenocytosine (εC) (**C**) in the amygdala (AMG) of sheep infused with the lower (KYNA 20–20 μg in total/day) and higher (KYNA 100–100 μg in total/day) dose of kynurenic acid into the third ventricle of the brain. Significance of differences: cd, *P* < 0.05; ab, *P* < 0.01; AB, CD, *P* < 0.001.

The repair activity of glycosylases for individual modified nucleobases differed between the treatment groups in individual brain structures (Figs. 5, 6, 7, 8). While both doses of KYNA significantly stimulated the efficiency of 8-oxoG excision in most tissues tested (Figs. 5A, 6A, 8A), there was an unexpected decrease in excision efficiency observed in the hippocampal CA3 field in response to both KYNA doses (Fig. 7A) compared to controls.

Similarly, both the lower and higher doses of KYNA significantly influenced the effectiveness of εA excision compared to the control group in all analyzed structures (Figs. 5B, 6B, 7B, 8B). Interestingly, the excision efficiency for εA in the POA was higher for lower dose of KYNA compared to the control and higher dose of KYNA (*P* < 0.05– < 0.001) (Fig. 6B). Both doses of KYNA increased the excision efficiency of εC in all structures (Figs. 5C, 6C, 7C, 8C) compared to controls however, in CA3 (Fig. 7C) and AMG (Fig. 8C), the effect of the lower dose was almost twice as high as that observed in the MBH and POA.

## Discussion

This study shows new data on the potential protective effects of KYNA in the CNS, focusing on the gene transcripts expression and functional activity of BER pathway enzymes in specific areas of the sheep brain, i.e., hypothalamus (including MBH and POA), hippocampus (CA3 field) and amygdala.

The brain, being highly susceptible to reactive oxygen species (ROS) due to its oxygen-dependent metabolism, elevated energy requirements, lipid-rich composition, and relatively limited antioxidant defenses, compared to other tissues, is particularly vulnerable to oxidative stress. In addition to enzymatic antioxidant protection of cells, specific glycosylases of the BER pathway identify and eliminate DNA base damage induced by both exogenous mutagens and internally generated harmful radicals. Despite the body's antioxidant defense systems, an imbalance between the production of reactive oxygen species and the repair of oxidative damage may lead to DNA mutations. Oxidative DNA damage and mutations, which can result from impaired DNA repair pathways, have been shown to be associated with cancer development and progression, as well as aging and age-related neurological disorders^30,31^. Fluctuations in KYNA production, particularly within the CNS, where it exerts potent neuromodulatory and neuroprotective effects, are believed to contribute to these adverse changes^32^. In the present study KYNA was infused into the third ventricle of the brain. Therefore, there was the possibility for KYNA to spread with the cerebrospinal fluid through the brain ventricular system (even countercurrent) to the adjacent structures such as hypothalamus (from the third ventricle), hippocampus and amygdala (from the lateral ventricle). It should be noted that ventricular system of the sheep’s brain, as in humans, includes the lateral ventricles, third ventricle, cerebral aqueduct, and fourth ventricle, which are filled with cerebrospinal fluid (CSF). According to the classical model, CSF production takes place mainly at the choroid plexuses located in the ventricles and their ependymal layer. Therefore, the CSF flow is directed caudally through the ventricles and the subarachnoid space toward the arachnoid villi, where it is absorbed into the venous blood^33^. The present study revealed a significant increase in OGG1 mRNA expression in response to both doses of KYNA across all analyzed brain structures. OGG1 is the main glycosylase that catalyzes the excision of 8-oxoG from duplex DNA when paired with cytosine, initiating base excision repair (BER)^34^. This process involves cleaving the glycosidic bond between 8-oxoG and sugar moiety, creating an abasic site^35^. Damage to 8-oxoG lesion can cause mutations and genomic instability if left unrepaired. The mammalian brain is composed of different types of cells, such as neurons and microglia, each harboring two separate genomes, one in the nucleus and the other in the mitochondria. According to Sheng et al., neurodegeneration caused by the accumulation of 8-oxoG lesions is a complex process that may occur in these distinct brain genomes^36^. In addition, our study demonstrated that the efficiency of 8-oxoG excision was also increased in response to infusions with both KYNA doses, however, it should be noted that the increase in 8-oxoG removal efficiency in the MBH, POA and AMG was higher in response to the lower KYNA dose. Interestingly, in most cases, the increase in enzyme activity reflected the high level of expression of their genes. It can be inferred that the stimulation of OGG1 mRNA expression by KYNA is consistent with its antioxidant effect^37^. Moreover, in a study by Roszkowicz-Ostrowska et al., KYNA infusion into the third ventricle of the sheep brain stimulated OGG1 mRNA expression in the CA1 region, while none of the KYNA doses significantly affected the efficiency of 8-oxoG excision compared to the control group^38^. The role of OGG1 in the elimination of 8-oxoG in the central nervous system (CNS) is not fully understood. Nonetheless, some studies have suggested that OGG1 deficiency may contribute to neurodegeneration, cognitive impairment and neuroinflammation. Therefore, OGG1 and the elimination of 8-oxoG may have important implications for preserving genomic integrity and neuronal health in the CNS. It is worth nothing that, the accumulation of 8-oxoG in the genome has been shown to induce cell death through a common mechanism found in mammals^39^.

Apart from 8-oxoG, ε-derived lesions of DNA bases, such as εA and εC, also have an equally high mutagenic potential. It has been established that both ε bases are recognized and cleaved by monofunctional DNA glycosylases. The first one of these is MPG, which typically repairs DNA bases damaged by alkylation, while the second glycosylase is TDG, responsible for repairing DNA damage resulting from deamination^40^. In the present study, a clear increase in MPG mRNA expression was observed in all brain structures examined following KYNA infusion into the third ventricle of the brain, while the expression of TDG mRNA increased only in response to the lower dose. It should be noted that TDG interacts with members of the nuclear receptor superfamily, including the androgen receptor, glucocorticoid receptor, progesterone receptor, vitamin D3 receptor, peroxisome proliferator-activated receptor, thyroid hormone and estrogen receptor (ER)^41–43^. Importantly, approximately half of the characterized TDG binding sites have been found to overlap with estradiol-mediated ER binding^44^. In addition, KYNA can also modulate ER phosphorylation through various kinases such as AKT and ERK, which play crucial roles regulating ER stability, localization, and function^45^. This suggests that KYNA may act as an endogenous estrogen-like compound capable of modulating the expression of estrogen-responsive genes. Therefore, KYNA may impact the interplay between estrogen and other signaling pathways in various physiological and pathological contexts. The intricate relationship between KYNA and TDG serves as a complex and dynamic regulator of estrogen signaling, with potential implications for understanding and treating diseases such as breast cancer, metabolic disorders, and neurodegeneration^46^.

Moreover, KYNA effectively induced the excision efficiency of εA and εC bases, however, for εA bases in the POA and εC in CA3 and AMG, the excision efficiency was higher with a lower dose infusion. Importantly, the lower excision efficiency obtained for the higher dose may be indicative of KYNA's own antioxidant properties^47^. On the other hand, the often noticeable higher effectiveness of a lower dose of acid in stimulating both the expression and activity of glycosylases may indicate a certain sensitivity threshold of cells to this compound. Roszkowicz-Ostrowska et al. also observed a dose-dependent increase in glycosylase gene transcripts expression and ε base excision efficiency in the hippocampal CA1 field in response to KYNA infusion^38^.

Finally, KYNA also increased the abundance of APE1 mRNA transcripts in the sheep brain structures. These findings are consistent with our previous study, where KYNA increased the expression of APE1 in the CA1 field of the hippocampus in a dose-dependent manner^38^.The precise mechanism by which KYNA modulate APE1 expression remains unclear, although it has been suggested to involve the activation of NMDA receptors and modulation of intracellular calcium levels. For example, APE1 is directly involved in the Ca^2+-^dependent downregulation of parathyroid hormone expression by binding to negative calcium response elements (nCaREs) present in the PTH promoter. APE1 is acetylated both in vivo and in vitro by the transcriptional coactivator p300, which is activated by Ca^2+.^ In contrast, acetylation at Lys6 or Lys7 increases APE1 binding to nCaREs^38^. Studies in rodents have shown that reduced APE1 levels increase cell sensitivity to oxidative stress^48^. Other study demonstrated that depletion of APE1 in cultured hippocampal and sensory neurons significantly sensitized cells H_2_O_2_-induced oxidative DNA damage, as evidenced by reduced cell viability, increased caspase-3 activity and histone H2AX phosphorylation^49^. In contrast to APE1 depletion, its overexpression was shown to induce a neuroprotective effect in dorsal root ganglion neurons exposed to cisplatin^50^. In addition, it has been found that it is the DNA repair function of APE1 that is crucial for the survival of post-mitotic cells exposed to oxidative stress conditions^50^.

In conclusion, the present study demonstrates that KYNA can stimulate the expression of certain genes encoding BER pathway enzymes, as well as affect their enzymatic activity in specific structures of the sheep brain. Our findings imply that, KYNA's ability to activate the BER system in the CNS may be related to its antioxidant properties.

### Software used in experiments

1. Primer3, version 4.1.0; The Whitehead Institute, Boston, MA, USA; https://primer3.ut.ee/.
2. The Rotor Gene 6000 software 1.7 containing Relative Expression Software (Qiagen GmbH, Hilden, Germany); software provided by the manufacturer.
3. STATISTICA, Stat Soft, Tulsa, OK, USA; https://www.statsoft.pl/statistica_13/.

## Data Availability

The data underlying this article will be shared on reasonable request to the corresponding author.
